# Navigating health challenges: the interplay between occupation-imposed movement restrictions, healthcare access, and community resilience

**DOI:** 10.1186/s12889-024-18817-y

**Published:** 2024-05-13

**Authors:** Oqab Jabali, Abed Alkarim Ayyoub, Shaden Jabali

**Affiliations:** 1 Language Center, Faculty of Humanities and Education Sciences, An-Najah National University, Nablus, Palestine; 2 Psychology and Counseling Department, Faculty of Humanities and Educational Sciences, An Najah National University, Nablus, Palestine; 3 Palestinian Ministry of Health, Ramallah, Palestine

**Keywords:** Community resilience, Health outcomes, Palestine, Road blocks, Transportation restrictions

## Abstract

**Background:**

Transportation plays a significant role in health, community resilience, and access to basic needs such as healthcare, social services, education, and job opportunities. Health and community resilience are, however, impacted by a multitude of complex and unequal factors, such as transportation restrictions exacerbated by the Israeli occupation. The goal of the research was to examine the intricate relationships that exist in Palestine between movement restrictions imposed by occupation, health outcomes, and community resilience.

**Method:**

A self-structured questionnaire, devised based on expert conversations and previous literature, was used in this descriptive, quantitative study to explore health and resilience outcomes. Age, gender, marital status, place of residence, and means of transportation were among the various factors that were utilized to describe the socio-demographics of the study sample (*n* = 202). The researchers employed stepwise multiple regression and descriptive statistics for the data analysis.

**Results:**

Study findings indicated that transportation restrictions have significant direct and indirect health consequences. A significant direct effect is observed, signifying a negative correlation between restrictions and health; increased transportation restrictions are consistently correlated with a decline in health. The study emphasized how place of residence affects health outcomes, with higher scores for campers and people living in green line areas. It also underscores that public means of transportation are found to be better for health outcomes than private cars.

**Conclusion:**

This study emphasized that roadblocks, checkpoints, and military incursions make it difficult for Palestinians to receive medical care, which has a detrimental impact on their health and well-being. It also underscores the need for significant reforms in Palestinian health and transportation systems to enhance infrastructure and healthcare access. The Palestinian Authority should invest in public transportation and community resilience programs to address transportation-related health issues, especially in villages, due to frequent settler attacks.

## **Introduction**

Transportation is crucial for economic growth, social connectivity, and access to essential and basic needs [1–3]. However, it also has a complex impact on public health outcomes, affecting aspects beyond physical mobility. Car-centric modes of transportation have led to increased global health concerns such as obesity, air pollution, climate change, and traffic accidents [4]. These issues have long-term implications for environmental sustainability and socioeconomic well-being and pose immediate risks to public health. The global prevalence of car-centric transportation systems has worsened urgent health issues, highlighting the need for sustainable transportation solutions [5].

While transportation is widely acknowledged to have negative health impacts beyond the direct effects of vehicle pollutants and accidents, exposure to conflict environments presents a separate yet equally significant risk to the physical and mental well-being of civilians [6, 7]. The disruptive and often traumatic nature of conflict situations can lead to a wide array of negative health outcomes, including but not limited to physical injuries, psychological distress, and long-term mental health disorders.

In addition, transportation systems play a major role in several environmental damage processes, such as habitat destruction, noise pollution, and biodiversity disturbance [8]. Such adverse environmental conditions have been associated with the development and aggravation of health conditions related to stress as well as an increase in illnesses linked to inactivity. Many chronic diseases, including type 2 diabetes, cardiovascular disease, dementia, and particular types of cancer, have been associated with sedentary lifestyles caused by an overdependence on motorized means of transportation [8].

However, transportation contributes to the burden of disease through road traffic injuries, air pollution, and physical inactivity. Road accidents have immediate health impacts and long-term consequences for families and communities. Air pollution from fossil fuel-driven vehicles is associated with respiratory and cardiovascular ailments, further exacerbating the burden of disease [9, 10]. Physical inactivity, a risk factor for noncommunicable diseases, is exacerbated by transportation policies [11]. The availability of transportation influences social determinants of health, including healthcare access and environmental well-being [12–16].

However, the impact of transportation extends beyond physical health concerns. While transportation systems contribute to the burden of disease through various factors, understanding how individuals cope with these challenges is equally crucial. Recent research has shown that resilience plays a pivotal role in shaping individuals’ responses to adversity and stressors, ultimately influencing subjective well-being and mental health outcomes.

According to recent research, there is a strong correlation between resilience and subjective well-being [17, 18]. More resilient people have been shown to have stronger coping mechanisms for difficult situations and are more capable of recovering from traumatic experiences [18–20]. Moreover, some studies have indicated that resilience functions as a precursor to some outcomes related to subjective well-being, including mental health [19] and general life satisfaction [17]. The ability to adjust effectively in the face of hardship, trauma, tragedy, threats, or substantial amounts of stress is known as resilience [21]. It has been recognized as a distinguishing characteristic that can impact a person’s overall health [22]. Greater life satisfaction, greater self-esteem, and fewer depression symptoms are related to greater resilience [23–25].

## Patient pathway in Palestine

In this intricate network of interactions between transportation, conflict, and health, the Palestinian healthcare system stands as a beacon of resilience and adaptability. The journey begins with prompt assessments at primary healthcare centers, ensuring seamless referrals to specialized care when needed. Comprehensive health insurance schemes such as the Palestinian Health Insurance (PHI) or private plans alleviate financial barriers, while meticulous follow-up appointments and community health worker support ensure continuity of care [17]. Crucially, mental health services are seamlessly integrated into the framework, recognizing and addressing the profound psychological toll of conflict-affected living.

However, bridging the chasm between theoretical ideals and stark realities in Palestine remains a formidable task. The ongoing conflict casts long shadows on healthcare access and delivery, underscoring the urgent need for holistic solutions that navigate the intricate interplay of transportation, conflict, and health [17]. As research delves deeper into these complexities, understanding the multifaceted nature of resilience becomes paramount. From attachment security to freedom of movement, resilience emerges as a linchpin in navigating adversity and fostering adaptive functioning amidst hardship. Secure attachments and robust coping mechanisms pave the way for greater resilience, offering a beacon of hope amidst the tumultuous landscapes of conflict and transportation challenges.

## Literature review

Numerous factors contribute to the influence of conflict situations on an individual’s well-being, and research indicates that different people are more or less susceptible to negative outcomes [17]. Some people are resilient and have adaptive coping strategies, whereas others are more prone to increased distress and impairment. Environmental and psychological elements are important in determining how people are affected by conflict environments. Environmental factors that have a substantial impact on the general health outcomes of impacted groups include resource scarcity, political violence, and abuses of fundamental liberties [17]. In addition, psychological traits such as stress management and emotional control have a significant impact on how resilient or vulnerable a person is in the face of hardship.

In light of the Israeli-Palestinian conflict, the importance of freedom of movement has drawn attention because it is thought to be detrimental to general well-being [26]. Furthermore, greater psychological consequences are linked to increased exposure to conflict, especially for Palestinians, who have more negative psychological impacts than Israelis [26]. Due to limited access to healthcare, movement restrictions such as roadblocks, checkpoints, and the Apartheid Segregation Wall have been found to negatively affect the physical health [27] and psychological well-being of Palestinians [28].

Attachment security is necessary to promote resilience and adaptive functioning in the face of disasters and hardships [29]. Secure attachment, characterized by optimistic ideas about oneself and others, promotes greater resilience. Securely attached individuals might exhibit strong emotional regulation, have adaptable coping mechanisms and are more likely to seek support from others in difficult situations. On the other hand, insecure attachment types are linked to lower resilience and make it difficult for a person to trust others, rely on themselves, and seek help [29].

Ongoing political violence perpetuates a cycle of humiliation and disruption for Palestinians, particularly at checkpoints and separation walls, exacerbating psychological distress [30, 31]. A 25-year study conducted from 1987 to 2011 revealed that, in comparison to those who only sometimes faced political violence, 12% of Palestinians living in locations with a higher concentration of Israeli military presence had long-term humiliation and worsened health, economic, political, and psychological functioning [31, 32]. Another study examined the wider effects of political unrest on health, observing that different health indicators are affected by movement restrictions, limited access to energy, and problems with checkpoints [33]. Additionally, restrictions imposed by Israel significantly impact Palestinians’ livelihoods, including their access to essential services such as healthcare and education [34, 35]. These constraints have led to increased rates of preterm birth, reduced postnatal care utilization, and heightened emotional and behavioral psychopathology among children in affected areas [36, 37].

Teenagers exposed to political violence demonstrate signs of posttraumatic stress disorder and depression, with violence also negatively impacting family and social functioning [38, 39]. Women’s PTSD symptoms are positively correlated with exposure to political violence, which contributes to intimate relationship violence [40, 41]. Overall, political violence exposure is associated with increased PTSD symptoms among women.

Resilience is essential for improving mental health and quality of life, especially for those who are facing ongoing, persistent conflict and violence, as is the case in Palestinian-occupied territories [42]. Resilience is defined as the capacity to deal with trauma effectively, accomplish positive goals in the face of adversity, and apply constructive problem-solving techniques [43]. Resilience in Palestinian culture is frequently associated with the idea of “sumud,” which means steadfastness or resilience [44, 45]. According to cross-national studies, the resilience of Palestinian cultures leads to improved psychological well-being and quality of life compared to those of their counterparts in more tranquil situations [46]. Furthermore, among adult Palestinians, resilience is favorably correlated with feelings of well-being and psychological functioning [47].

Resilience among Palestinians is a cornerstone of their collective experience, showcasing their remarkable ability to navigate and surmount a myriad of challenges, including the enduring impact of political violence [41]. This resilience is not merely a passive trait but is actively cultivated through interventions aimed at bolstering the well-being and mental health of children and adolescents affected by the pervasive effects of war and armed conflict [42]. Furthermore, recent investigations have delved deeper into the complex interplay between social skills, grief, and resilience among Palestinian university staff members and students, particularly within the context of the ongoing COVID-19 pandemic, shedding light on the nuanced dynamics of resilience in the face of adversity [43]. Moreover, scholarly examinations of the social ecology of resilience, encapsulated by the concept of Sumud, provide valuable insights into the interconnectedness of Palestinian communities and their collective capacity to endure and thrive amidst challenging circumstances, underscoring the multifaceted nature of resilience within Palestinian society [44].

The research gap addressed by this study lies in the limited understanding of the multifaceted relationships between resilience, health, and transportation restrictions within the Palestinian context. While previous research has acknowledged the immediate impact of transportation limitations on health outcomes, comprehensive analyses of the indirect effects of these restrictions on community resilience are lacking. By exploring these complex interactions, this study seeks to bridge this gap by providing insights into how transportation constraints not only directly affect individual health but also indirectly influence community resilience, thereby contributing to a more nuanced understanding of the health challenges faced in the Palestinian context.

Based on the identified gaps in the literature and the research questions posed, we can formulate the following hypotheses:

H1: Transportation restrictions have a negative impact on health outcomes among affected populations. Specifically, we hypothesize that individuals living in areas with greater transportation restrictions will report poorer physical and mental health than those with fewer restrictions.

H2: Transportation restrictions have a direct impact on community resilience. We predict that communities facing greater transportation limitations will exhibit lower levels of resilience, as measured by their ability to adapt and respond to challenges effectively.

H3: Community resilience mediates the relationship between transportation restrictions and health outcomes. We hypothesize that transportation restrictions indirectly affect health outcomes by influencing community resilience. Specifically, we expect that communities with higher levels of resilience will demonstrate better health outcomes despite facing transportation limitations.

H4: There are statistically significant differences among respondents based on their demographic characteristics in terms of their perceptions of transportation restrictions, community resilience, and health outcomes. We predict that demographic factors such as age, gender, socioeconomic status, and geographic location will influence individuals’ perceptions of transportation restrictions, their level of resilience, and their reported health outcomes.

## Statement of the problem

Transportation infrastructure is especially important in Palestine, an area with distinct geopolitical concerns and socioeconomic imbalances [35]. Transportation networks have a significant impact on public health outcomes in addition to being necessary for enabling mobility and granting access to necessary services [41]. In Palestine, the relationship between mobility and health is complicated and uneven, ranging from restrictions placed by political boundaries to the harmful health impacts of air pollution and accidents caused by moving vehicles [45].

Restrictions on transportation have become a major issue in Palestine, having a substantial impact on the population’s resilience and overall health [42]. These restrictions, imposed in various forms, pose barriers to accessing essential services, including healthcare, education, and employment opportunities [42, 45]. In addition to negatively affecting people’s health outcomes, these limitations reduce communities’ ability to cope with and recover from difficult situations [43, 44, 46].

Living conditions in Palestinian territories vary significantly due to the complexities of occupation and conflict [34, 38]. Area A, under full Palestinian control, consists mainly of urban centers with dense populations characterized by overcrowding, limited access to basic services, and inadequate infrastructure. Access to healthcare services can be strained due to resource shortages and restrictions on movement [39]. Area B, designated under the Oslo Accords as under Palestinian civil control but with joint Israeli-Palestinian security, also faces similar challenges due to infrastructure, services, and healthcare access [38].

Area C, under full Israeli control, encompasses the majority of the West Bank, including rural and agricultural regions. Palestinians face significant difficulties accessing essential services such as healthcare, education, and water resources due to restrictions on development and construction [45]. Refugee camps, primarily located in Areas A and B, house Palestinians displaced during the 1948 Arab-Israeli War and their descendants [39]. Movement within and outside of refugee camps can be restricted by Israeli military checkpoints, affecting access to healthcare facilities and essential services. Palestinians living in Green Line areas, known as pre-1967 borders, still face systemic discrimination and socioeconomic disparities, hindering access to healthcare facilities and services [39, 48].

Every day, Palestinians who commute within and to the West Bank, regardless of their nationality or residency status, must navigate Israeli checkpoints as part of their daily routine. This includes Palestinians living in the Green Line areas, who are often treated as West Bank residents in terms of checkpoint procedures. These checkpoints pose significant challenges and delays for Palestinians, affecting their ability to travel freely within their own territories and impacting various aspects of their daily lives, including access to education, healthcare, employment, and social activities. The experience of crossing checkpoints is a shared reality for Palestinians, underscoring the pervasive impact of occupation-imposed movement restrictions on individuals’ lives and livelihoods.

**Fig. 1:**
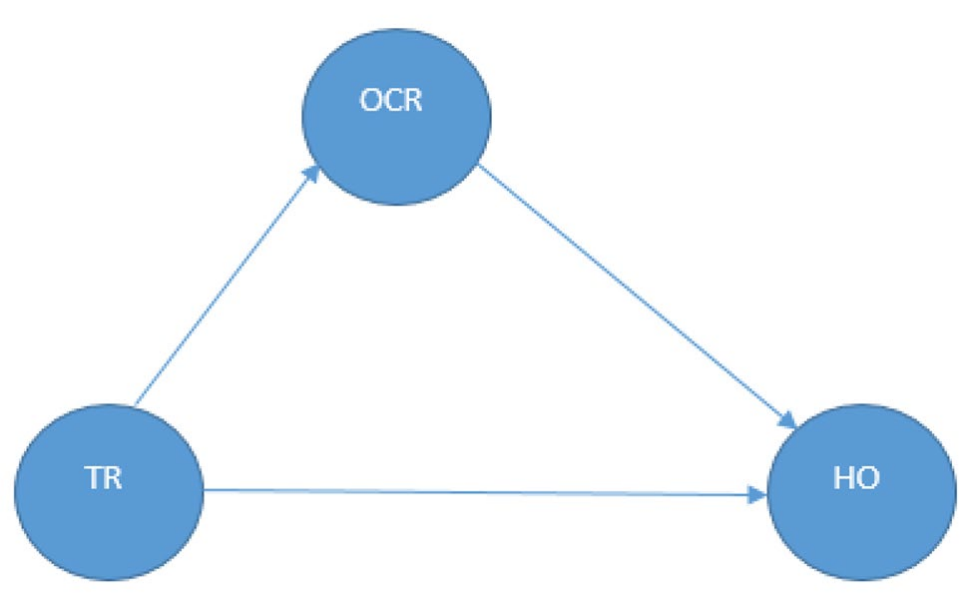
Hypothesized study model. TR: Transportation Restrictions, HO: Health Outcomes, OCR: Outcomes of Community Resilience

This study provides a model for examining the complex interactions between resilience, health, and transportation restrictions in the Palestinian context. The authors aim to show that restrictions on transportation have multiple effects: primarily, they have an immediate impact on health results, which can be seen as negative consequences for people’s health situations. Second, transportation restrictions also have an indirect effect on health outcomes by impacting community resilience. This model, as shown in Fig. 1 below, enables us to investigate both the direct impact of movement restrictions on well-being and the indirect influence of transportation restrictions on health outcomes through their effect on community resilience. The researchers endeavored to answer the following questions:

- How do transportation restrictions impact health outcomes among affected populations?
- How do transportation restrictions impact community resilience?
- How does community resilience impact health outcomes through its mediating role?
- Are there statistically significant differences among respondents based on their demographic characteristics in terms of their perceptions of transportation restrictions (direct variable), community resilience (indirect variable), and health outcomes (dependent variable)?

## Methods

In this section, we detail the methodology employed to collect and analyze data for our study on the impact of transportation restrictions on health and resilience. The survey instrument utilized in this research was disseminated through Google Forms and distributed directly to the email addresses of Palestinian university employees and staff members in February 2024. The survey remained open for a duration of two weeks to allow participants ample time to respond. A total of 202 valid responses were obtained, forming the basis of our quantitative analysis. This study adopts a descriptive research approach to examine [research objectives or variables of interest]. The following subsections provide a comprehensive overview of the survey administration process, data collection procedures, and analytical techniques employed in this study.

### Data analysis

The analysis used frequencies to show the percentages for every level of the demographic variables, promax exploratory factor analysis to adapt the tool items to Palestinian university employees and staff members, partial least squares structural equation modeling to show the direct and indirect effects of the constructs, and stepwise multiple regression techniques to explore the impact of demographic variables as moderators between the constructs and the dependent variable.

### Participants

The sample encompasses university employees and staff members from diverse backgrounds, reflecting variations in qualifications, income levels, age groups, and ownership of vehicles, among other pertinent factors. These differences in demographics and socioeconomic characteristics contribute to a comprehensive understanding of the impact of transportation restrictions on health outcomes and community resilience within the Palestinian context. By including individuals with varying qualifications, income levels, and transportation modes, this study can effectively explore the nuanced effects of transportation limitations on different segments of the population and assess how community resilience mediates these impacts across diverse demographic groups.

For partial least squares structural equation modeling (PLS-SEM), choosing the right sample size is crucial to ensuring the validity and precision of the findings. The sample size in PLS-SEM searches is variable and depends on various aspects, including the degree of statistical power needed, the number of latent variables and indicators, the complexity of the model, and the magnitudes of the effects. A minimum sample size of 100–200 observations is recommended by some researchers, while a sample size-to-indicator ratio of at least 5:1 or 10:1 is advised by others. We employ 202 observations, although a large sample size of 110 is required for our investigation because we use 11 indicators.

### Study tools

The study uses a survey instrument to gather data on the impact of Israeli occupation on Palestinian communities. It focuses on three main domains: transportation restrictions, health outcomes, and outcomes on community resilience. Demographic variables are used to represent respondents’ backgrounds. The Transportation restrictions domain assesses participants’ agreement with the impact of the Israeli occupation on movement and essential services. The Health Outcomes domain examines the effects of restrictions on physical and mental well-being, including accident risk, delays in accessing critical medical care, and exposure to environmental hazards. The Outcomes on Community Resilience domain evaluates Palestinian communities’ resilience in response to restrictions, considering factors such as unity, recovery, belonging, and leadership confidence.

The respondents rated their agreement using a five-point Likert scale. The questionnaire was developed after detailed revision by experts and an investigation of previous literature on the impact of transportation on health. Nine expert arbitrators examined the pool of 39 items, evaluating their correlation with the total number. The researchers ignored all the items that had a correlation less than 0.60. In the end, the survey was limited to thirty items, with 10 items for every domain.

## Results

Different variables are used to characterize the sociodemographics of the sample population, as shown in Table 1. With 46% of the respondents being male and 54% being female, the gender breakdown indicates a fairly balanced representation. Regarding social status, 32.2% of people reported being single, while the majority (67.8%) were married. The distribution of educational levels shows that a large majority (52.5%) have a bachelor’s degree, followed by a high school degree (18.8%) and a master’s or doctoral degree (9.9%). A total of 36.6% of the population lives in cities, 52.5% in villages, and 10.9% in refugee camps. There is diversity in the political areas; area A has the largest percentage (58.9%). The age distribution showed that the majority (64.9%) of people were between the ages of 18 and 24, with fewer people in the other age groups. The majority of modes of mobility are buses or taxis (55%), followed by private cars (33%) and bicycles or motorbikes (12%).

**Table 1:** Demographic characteristics

Variable	Levels	*N*	%
Gender	Male	93	46
	Female	109	54
Social status	Married	137	67.8
	Single	65	32.2
Education level	High school (Tawjihi)	38	18.8
	Vocational certificate	19	9.4
	Diploma	19	9.4
	Bachelor degree	106	52.5
	Master’s or doctoral degree	20	9.9
Place of residence	City	74	36.6
	Village	106	52.5
	Camp	22	10.9
Political area	A	119	58.9
	B	38	18.8
	C	23	11.4
	Green line	22	10.9
Age	18–24 years	131	64.9
	25–34 years	25	12.4
	34–45 years	23	11.4
	More than 45 years	23	11.4
Means of Transportation	Private car	67	33
	Taxi or Bus	111	55
	Bicycle or motorcycle	24	12

The analysis used exploratory factor analysis (EFA) to determine the underlying structures of participants’ perspectives on the impact of transportation constraints on health and resilience. A parallel analysis with main component extraction and oblique promax rotation was performed using JASP version 18.3. Significant connections between survey items and their factors were determined by loadings that exceeded 0.4, resulting in the identification of 11 survey items classified into three main domains (transportation restrictions, outcomes on health, and outcomes on community resilience), explaining 72.9% of the observed variation. Factorial validity and reliability were utilized, as shown in Table 2, to determine whether the study tool fit the variables under investigation. While reliability ensures consistent and stable findings under different conditions or settings, validity guarantees that the tool items appropriately represent the intended theoretical conceptualization.

**Table 2:** Results of factor analysis for the survey domains along with the total scale

Items	Transportation Restrictions	Outcomes on Community Resilience	Health Outcomes	Variance
A6)The presence of checkpoints and road closures by Israeli forces significantly restricts movement, creating inconvenience and lengthening travel times for Palestinians.	0.917			0.26
A2)Military patrols and checkpoints have hindered my attendance at both indoor and outdoor gatherings.	0.875			
A1)Physical barriers such as concrete blocks impede ambulances and emergency vehicles, leading to delays in medical emergencies and compromising health outcomes.	0.849			
A8)Transportation restrictions limit access to recreational facilities and hinder opportunities for exercise and leisure.	0.743			
C9)My confidence in our community’s resilience is positively affected by movement restrictions.		0.941		0.246
C8)Restrictions on movement positively affect my satisfaction with the level of social support available to me from my community.		0.861		
C3)Restrictions on movement positively influence my confidence in the leadership and governance structures within my community.		0.725		
B7)Limited or delayed access to restroom facilities during travel cannot prevent urinary tract infections or other health issues.			0.899	0.223
B6)Exposure to tear gas or sewage water while traveling cannot alleviate respiratory issues or exacerbation of existing conditions.			0.867	
B8)Prolonged exposure to extreme temperatures or colds while waiting at checkpoints cannot prevent weather-related illnesses.			0.827	
B9)The physical strain of enduring long waits at checkpoints cannot improve chronic pain or musculoskeletal issues.			0.812	
Total				0.729

Note. The applied rotation method was promax

The factor loadings presented in the study indicate the strength of the relationships between transportation restrictions and various outcomes, community resilience, and health outcomes. High factor loadings, such as 0.917 for movement restrictions affecting inconvenience and longer travel times, suggest a significant association between these restrictions and the observed outcomes. Similarly, loadings ranging from 0.725 to 0.941 for factors such as confidence in community resilience and satisfaction with social support demonstrate the substantial influence of transportation limitations on community dynamics. Additionally, factor loadings ranging from 0.812 to 0.899 for health-related factors, including access to restroom facilities and exposure to environmental hazards, underscore the impact of transportation restrictions on health outcomes. Overall, these loadings provide quantitative evidence of the complex interplay between transportation barriers and various aspects of Palestinian life, highlighting the need for comprehensive interventions to address these challenges effectively.

To answer the first three questions (How do transportation restrictions impact health outcomes among affected populations? How do transportation restrictions impact community resilience? How does community resilience impact health outcomes through its mediating role? The researchers conducted structural equation modeling (SEM), as shown in Tables 3, 4 and 5.

To estimate associations between observable and implicit variables, structural equation modeling (SEM) was used, and the results are shown in Table 3.

**Table 3:** Structural Model Results for the survey domains

Domain	item	Estimate	*p*	SRW	Cronbach’s α	CR	AVE
Outcomes on health	B7	1		0.825	0.86	0.89	0.84
	B6	0.964	< 0.001	0.817			
	B8	0.993	< 0.001	0.766			
	B9	0.959	< 0.001	0.802			
Outcomes on Community Resilience	C9	1		0.853	0.79	0.82	0.75
	C8	0.98	< 0.001	0.73			
	C3	0.843	< 0.001	0.774			
Transportation Restrictions	A6	1		0.756	0.81	0.83	0.8
	A2	0.84	< 0.001	0.853			
	A1	0.842	< 0.001	0.83			
	A8	0.932	< 0.001	0.758			
Total					0.89		

The results in the table above show that the Outcomes on Health domain exhibits positive associations with all items (B7, B6, B8, and B9), with a Cronbach’s alpha (α) of 0.86 and a composite reliability (CR) of 0.89. These items explained 84% of the variance in health outcomes. In the Outcomes on Community Resilience domain, items C9, C8, and C3 demonstrated positive associations, with SRW values ranging from 0.843 to 0.98. The domain has a Cronbach’s alpha of 0.853 and a composite reliability of 0.79, explaining 75% of the variance. Similarly, the Transportation Restrictions domain shows positive relationships between items A6, A2, A1, and A8, with SRW values ranging from 0.756 to 0.932. This domain has a Cronbach’s alpha of 0.81 and a composite reliability of 0.83, accounting for 80% of the variance in the restriction construct.

To determine whether the hypothesized relationships accurately account for the observed data and assess the model’s alignment with these data, model fit indices are used, and the results are presented in Table 4.

**Table 4:** Model fit indices for the structural equation model

Index	Value
Chi square (χ2)	χ2 (41) = 85.8, *P* = 0.00
χ2/df	2.1
Comparative Fit Index (CFI)	0.98
T-size CFI	0.97
Tucker‒Lewis Index (TLI)	0.97
Bentler-Bonett Nonnormed Fit Index (NNFI)	0.96
Bentler-Bonett Normed Fit Index (NFI)	0.97
Bollen’s Relative Fit Index (RFI)	0.94
Bollen’s Incremental Fit Index (IFI)	0.97
Relative Noncentrality Index (RNI)	0.97
Root mean square error of approximation	0.055

The structural equation model fit indices reveal key findings: the chi-square test (χ2) indicates a significant difference between the observed and expected covariance matrices (χ2 (41) = 85.8, *p* = 0.00), with a χ2/df ratio of 2.1, suggesting an acceptable fit. The comparative fit index (CFI), Tucker‒Lewis index (TLI), and Bentler–Bonett normalized fit index (NFI) exceeded 0.95, indicating good fit. The Bentler-Bonett Nonnormed Fit Index (NNFI), Bollen’s Incremental Fit Index (IFI), and Relative Noncentrality Index (RNI) are approximately 0.97, suggesting satisfactory fit. The Bollen Relative Fit Index (RFI) was 0.94, which is acceptable. The root mean square error of approximation (RMSEA) is 0.055, which is below 0.08, indicating good model-data fit.

**Fig. 2:**
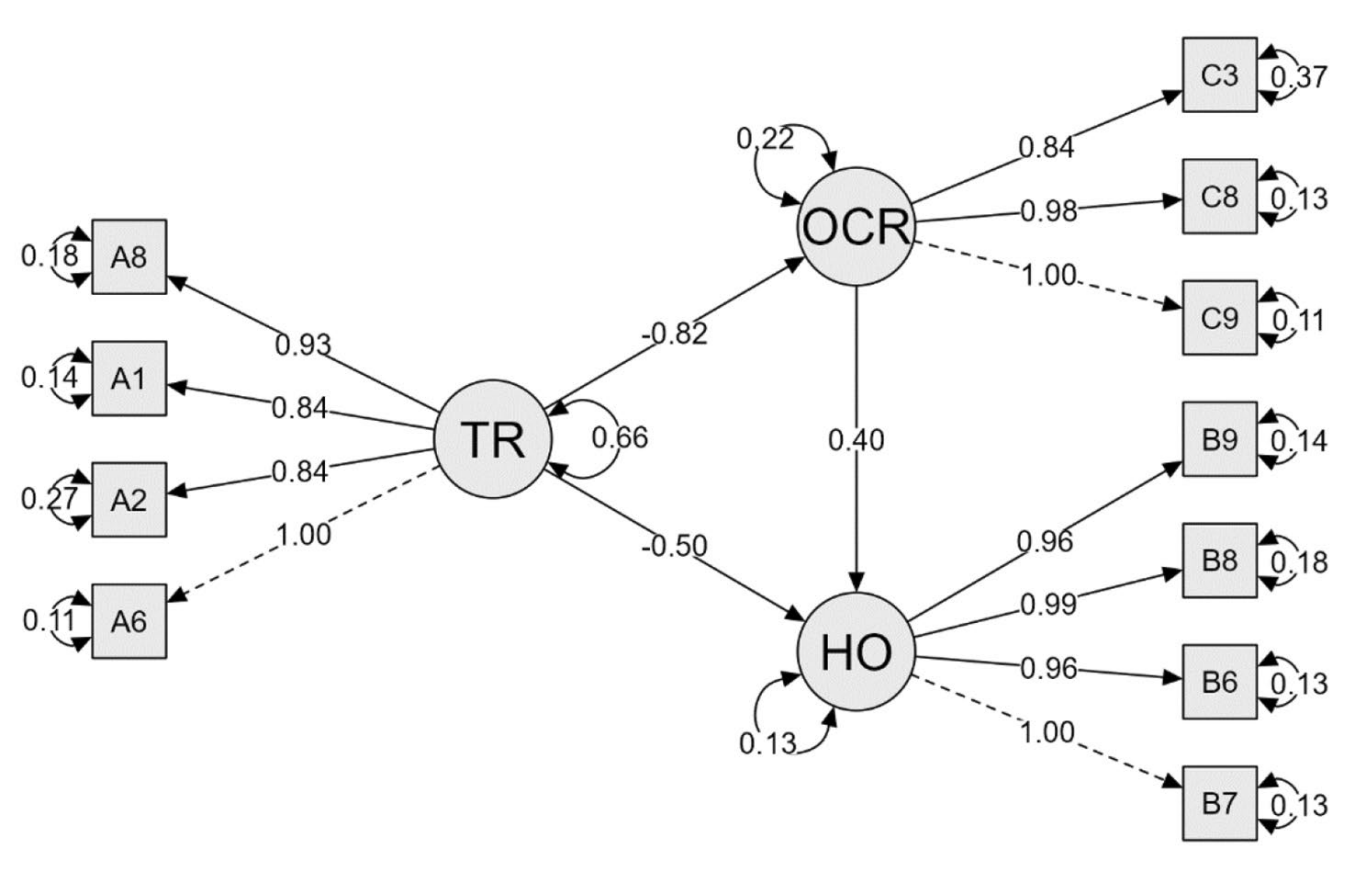
Model inner and outer variables

The strength and direction of the links between the variables in the structural equation model (SEM) are evaluated using causal path properties, as shown in Fig. 2.

The figure shows significant direct and indirect effects of transportation restrictions (TRs) on health outcomes (HOs). First, the direct effect (TR → HO) is estimated at -0.50, indicating a strong negative association between transportation restrictions and health outcomes, suggesting that as transportation restrictions increase, health outcomes tend to worsen. This direct effect is statistically significant, as evidenced by the z value of -8.63 and *p* < 0.001. Second, the indirect effect (TR → OCR → HO), mediated through outcomes on community resilience (OCR), is estimated at -0.33, implying that transportation restrictions indirectly influence health outcomes via their impact on community resilience. This indirect effect is also statistically significant, with a z value of -9.5 and a *p* value of less than 0.001, as shown in Table 5. Considering both direct and mediated pathways, the total effect of transportation restrictions on health outcomes is estimated at -0.83, indicating the combined influence of transportation restrictions on health outcomes. This total effect is statistically significant, supported by a z value of 9.21 and *p* < 0.001.

**Table 5:** Estimated Effects of Transportation Restrictions on Health Outcomes Mediated by Outcomes on Community Resilience

Effect		Estimate	z value	*P*
Direct	TR→ HO	-0.50	-8.6.3	< 0.001
Indirect	TR → OCR → HO	-0.33	-9.5	< 0.001
Total		-0.83	9.21	< 0.001

To answer the first three questions (How do transportation restrictions impact health outcomes among affected populations? How do transportation restrictions impact community resilience? How does community resilience impact health outcomes through its mediating role? The researchers calculated the means and standard deviations via stepwise multiple regression, as shown in Tables 6 and 7.

**Table 6:** Mean scores and standard deviations for the three domains across the demographic variables

Variable and levels		Transportation Restrictions		Outcomes on Resilience		Outcomes on Health	
		M	S D	M	S D	M	S D
Gender	Male	4.10	0.88	2.03	0.91	2.02	0.87
	Female	4.61	0.55	1.53	0.63	1.46	0.62
Marital status	Married	4.61	0.54	1.48	0.62	1.46	0.58
	Single	3.88	0.92	2.34	0.86	2.27	0.90
Education	High school (Tawjihi)	4.66	0.45	1.44	0.56	1.45	0.62
	Vocational certificate	3.20	0.94	3.02	0.53	2.87	0.80
	Diploma	3.64	0.72	2.53	0.94	2.21	0.82
	Bachelor degree	4.67	0.45	1.45	0.57	1.45	0.53
	Master’s or doctoral degree	4.11	0.83	2.07	0.65	2.13	0.89
Place of residence	City	4.56	0.61	1.50	0.70	1.47	0.66
	Village	4.38	0.82	1.75	0.78	1.71	0.76
	Camp	3.73	0.65	2.70	0.61	2.61	0.77
Political Area	A	4.52	0.56	1.59	0.71	1.59	0.70
	B	4.74	0.35	1.42	0.42	1.39	0.48
	C	4.41	0.59	2.13	0.87	1.95	0.85
	Green line	2.91	0.79	2.88	0.69	2.75	0.82
Age	18–24 years	4.63	0.50	1.47	0.59	1.45	0.55
	25–34 years	4.41	0.75	1.71	0.92	1.73	0.86
	34–45 years	3.83	0.76	2.55	0.69	2.17	0.69
	More than 45 years	3.43	1.03	2.67	0.72	2.79	0.92
Means of Transportation	Private car	3.21	0.74	2.94	0.51	2.64	0.65
	Taxi or Bus	4.65	0.46	1.44	0.54	1.45	0.53
	Bicycle or motorcycle	4.34	0.79	1.87	0.84	1.83	0.93

These results indicate slight variations rather than significant differences across different demographic factors in perceptions of transportation restrictions. Place of residence can significantly influence individuals’ exposure to transportation restrictions. The mean scores for “Place of Residence” (City: M = 4.56, SD = 0.61; Village: M = 4.38, SD = 0.82; Camp: M = 3.73, SD = 0.65) demonstrated significant differences, reflecting varied levels of transportation restrictions and potential disparities in health outcomes among residents. Political areas may experience differing levels of transportation restrictions due to security concerns and government policies. The ean scores for political area (A: M = 4.52, SD = 0.56; B: M = 4.74, SD = 0.35; C: M = 4.41, SD = 0.59; green line: M = 2.91, SD = 0.79) reveal significant differences, reflecting varying levels of transportation restrictions and their implications for health outcomes. The mean scores for “political area” (A: M = 4.52, SD = 0.56; B: M = 4.74, SD = 0.35; C: M = 4.41, SD = 0.59; green line: M = 2.91, SD = 0.79) demonstrate significant disparities, reflecting the diverse levels of transportation restrictions within different areas.

Significant disparities in resilience levels are evident across various demographic and contextual factors. Vocational certificate holders demonstrated the highest mean resilience score of 3.02, indicating strong resilience among this group, while bachelor’s degree holders exhibited the lowest mean score of 1.45, suggesting variability in resilience within this category. Among the different places of residence, camp residents had the highest mean resilience score of 2.70, indicating relatively high resilience levels, whereas city residents had the lowest mean resilience score of 1.50, with notable variability within urban areas. Residents in the “green line” area reported the highest mean resilience score of 2.88, suggesting consistent resilience levels, whereas those in Area B had the lowest mean score of 1.42, indicating less variability. These findings underscore the diverse resilience experiences influenced by education, place of residence, and political area.

Finally, the results in Table 6 above demonstrate that health perceptions vary based on various demographic factors. Compared with females, male respondents tended to report higher mean scores for health outcomes, suggesting potential differences in health perceptions. Single individuals also reported higher mean scores for health outcomes than did married individuals, suggesting that marital status may influence health perceptions. Vocational certificate holders reported higher mean scores for health outcomes than did those with other education levels. Holders with high school certificates and bachelor’s degrees reported more health outcomes. Compared with city dwellers, villagers reported slightly higher mean scores for health outcomes. Residents of political area B reported lower mean scores for health outcomes than did those in other areas, suggesting variations in health perceptions across different geographical regions. Older individuals, particularly those over 45 years old, reported higher mean scores for health outcomes than younger individuals did. In addition, compared to taxis, buses, bicycles, or motorcycles, private car users reported higher mean health outcome scores, suggesting that access to private transportation may influence health perceptions. To determine whether these disparities are statistically significant, the researchers used multiple regression.

To answer question (4- Are there statistically significant differences among respondents based on their demographic characteristics in terms of their perceptions of transportation restrictions (direct variable), community resilience (indirect variable), and health outcomes (dependent variable)? ) Multiple regression models were used to determine whether the differences were statistically significant, and the results are displayed in Table 7.

**Table 7:** Results of Multiple Regression Analysis: Assessing Predictors of Health Status

Model		*R* ^2^	B	t	*P*	F	*P*
1	(Constant)	0.792	5.31	26.722	< 0.001	336.486	< 0.001
	TR		-0.821	-18.34	< 0.001		
2	(Constant)	0.836	3.29	9.545	< 0.001	230.559	< 0.001
	TR		-0.515	-8.583	< 0.001		
	OCR		0.39	6.862	< 0.001		
3	(Constant)	0.84	3.152	9.098	< 0.001	158.617	< 0.001
	TR		-0.516	-8.682	< 0.001		
	OCR		0.353	6.053	< 0.001		
	PLACE		0.117	2.267	< 0.001		
4	(Constant)	0.844	3.001	8.529	< 0.001	121.855	< 0.001
	TR		-0.536	-8.964	< 0.001		
	OCR		0.352	6.083	< 0.001		
	PLACE		0.128	2.481	< 0.001		
	Transportation		0.1	2.026	< 0.001		

The above table demonstrates that all the models show that transportation restrictions (TRs) have a constant negative coefficient, meaning that when these constraints rise, health tends to decline. These connections are highly significant (*p* < 0.001). The outcome of resilience (OCR) usually has a positive coefficient, suggesting that higher health levels are correlated with better resilience outcomes, supported by highly significant positive associations (*p* < 0.001). The study findings also show that the persistent positive coefficient of place of residence (PLACE) suggests that living in villages is associated with better health than living in cities and that staying in camps is better than staying in villages or cities, with highly significant positive associations (*p* < 0.001). Furthermore, the coefficient of 0.1 with significant t- and *p* values shows that the transportation mode also substantially impacts health. Using public means of transportation is better than traveling on bikes or in private cars in Palestine.

## Discussion

The study findings underscore the multifaceted impacts of transportation restrictions on health outcomes, revealing both direct and indirect effects. Primarily, this research revealed a significant direct effect, indicating a negative correlation between transportation limitations (TLs) and health status. As TLs increase, there is a consistent decline in health outcomes, emphasizing the detrimental impact of restricted mobility on the population’s well-being. Additionally, the study revealed an indirect effect wherein limitations on transportation exert an influence on health outcomes through their impact on community resilience. This suggests that bolstering resilience can mitigate the adverse effects of transportation constraints on health, highlighting the crucial role of community-level resilience in combating health challenges.

Furthermore, the research findings align with the real-world challenges faced by Palestinians in navigating transportation restrictions, which manifest both directly and indirectly in health outcomes. Directly, the study revealed a negative association between transportation limitations and Palestinians’ ability to access essential healthcare services. Barriers such as road closures and authorization requirements impede individuals’ access to hospitals, clinics, and medical facilities, resulting in delayed or denied medical care. This restricted access exacerbates existing health conditions and contributes to the deterioration of overall health outcomes among Palestinians.

Moreover, the study findings resonate with previous research highlighting the adverse health impacts of conflict situations, which are exacerbated by limitations on movement. Studies [6, 7] have documented the significant stressors posed by conflict, leading to poorer psychological outcomes, particularly among Palestinians. The study’s identification of restricted access to healthcare due to roadblocks and checkpoints further underscores the complex interplay between conflict-related challenges and health outcomes. This elucidates the nuanced nature of the repercussions stemming from transportation restrictions in conflict-affected regions, emphasizing the urgent need for comprehensive interventions to address these interconnected issues.

Regarding the indirect impact of limitations in transportation on resilience, the study showcases the adaptive strategies Palestinian communities employ in dealing with constraints in transportation on health. Despite encountering obstacles in accessing healthcare, they rally resources, assistance networks, and initiatives in the community to tackle health issues [44]. This resilience enables them to endure and alleviate the repercussions of transportation constraints on health outcomes, underscoring the significance of community resilience in tackling health obstacles [46, 47]. The evidence indicates a robust link between resilience and individual well-being [17]. Individuals with high resilience exhibit superior coping mechanisms amid adversity and enhanced recovery from traumatic events [18]. Resilience also precedes a variety of subjective well-being results, encompassing mental wellness and general life contentment [17, 19, 20].

Significant differences in mean resilience scores were noted among inhabitants based on their residence and political region, with camp inhabitants and individuals in the “Green Line” district displaying relatively higher resilience scores than other groups. Refugee camps exhibit elevated resilience scores owing to the assistance systems offered by UNRWAs and NGOs. These entities provide emotional and social assistance, communal initiatives, and financial aid to aid inhabitants in managing challenges and navigating difficult situations. Despite political intricacies, these regions often enjoy superior access to resources, infrastructure, and socioeconomic opportunities. Residents in “green line” regions frequently experience enhanced living standards, better healthcare amenities, and increased access to education and work. The existence of supportive infrastructure, steady administration, and the availability of communal services in the “Green Line” region contribute to resilience by providing essential resources and opportunities for prosperity in the face of adversity. The constant political and socioeconomic environment also fosters a sensation of security and empowerment among inhabitants, further boosting their capacity to address issues.

Moreover, the place where one resides plays a crucial role, indicating that residing in a village is associated with better health compared to city living and that staying in a camp is more favorable than living in a village or city. The statistical significance of these associations (*p* < 0.001) underscores the impact of home location on health results. Despite being situated within major cities, camps may provide superior health outcomes due to their enclosed environment, and UNRWA-operated medical clinics are mainly situated within these camps. Camps in Palestinian regions are smaller, with fewer inhabitants in contrast to villages. Despite their limited size, they have adjusted to various constraints, including transportation limitations within and outside their vicinity [44–46]. Residents of camps have devised coping mechanisms and resilient strategies to address these obstacles [47]. For instance, they can establish volunteer-run transportation systems to aid individuals in accessing crucial services such as medical appointments. They can also create community-based health projects such as mobile clinics or telemedicine programs to bridge the healthcare accessibility gaps arising from transport restrictions.

In Palestine, villages are becoming increasingly self-sufficient in terms of services, markets, and hospitals. The difficulties presented by mobility restrictions and checkpoints may be the driving force behind this development, as they encourage local people to develop creative solutions to overcome obstacles [34]. Village residents may benefit from easier access to medical facilities and services, which could improve their health. Due to regular settler violence and attacks, which are frequently caused by Israeli expansion and control over the Palestinian territory, the safety and security of Palestinian village residents are serious issues [34, 35]. These assaults disturb everyday life, produce a hostile atmosphere, and have an impact on one’s physical and emotional well-being [17, 26–28]. These weaknesses are made worse by ongoing danger and the absence of security forces, which lead to a sense of insecurity and instability.

Checkpoints and other barriers erected by Israeli authorities frequently split Palestinian cities, making it difficult for locals to access basic amenities such as medical facilities, particularly when referring patients to Israeli hospitals that admit complex cases or to hospitals in Ramallah and Jerusalem. Compared to people living in villages or camps, this may result in worse health outcomes. The transportation limitations of Israeli authorities have a substantial influence on socioeconomic, humanitarian, and health-related difficulties in Palestinian cities, including Jenin, Tulkarm, and Nablus [42]. These regulations hurt companies and stifle economic progress by impeding the movement of products and services. Additionally, they worsen humanitarian issues by making it more difficult for Palestinians to access basic services such as work, healthcare, and education due to protracted lines and arbitrary closures at checkpoints [38, 39].

Residents experience feelings of alienation and isolation as a result of these limitations, which can contribute to social disturbance, fragmentation, and isolation. The Segregation Wall has a major impact on patients’ ability to obtain healthcare, making it more difficult for them to reach clinics or hospitals. This exacerbates health disparities and mortality [12, 13, 41]. The psychological health of the populace is also impacted by ongoing military invasions into these cities, as well as the existence of checkpoints and uncertainty.

Finally, the study’s results show that a person’s mode of transportation has a large influence on their health. In Palestine, using public transportation is found to be more healthful than riding a bike or driving a private vehicle. This finding highlights the need for easily available and well-functioning transportation systems for improving public health. Private vehicles, which must contend with Israeli military checkpoint closures, delays, and detours, are particularly affected by these limitations. The difficulties experienced by Palestinian drivers are made worse by settler attacks, particularly in regions near illegal Israeli settlements where harassment and acts of violence are frequent [28, 34, 35]. However, public transportation—such as buses and taxis—may be able to go through specific routes or receive exceptions at specific checkpoints, providing Palestinian commuters with a more dependable and secure means of transportation.

For Palestinian communities, public transportation alternatives offer a lifeline amidst the complexity of the Israeli occupation and continuous conflict, even though they face their own set of difficulties. In general, public transportation options endure fewer disruptions and delays than private cars. Furthermore, bus and taxi drivers are more likely than individual private car owners to have firsthand knowledge of safe routes due to their experience and social networks in the community. This knowledge allows them to bypass checks and steer clear of potentially hazardous locations. The aforementioned proficiency enhances the robustness of public transportation networks in Palestinian urban areas, guaranteeing uninterrupted availability of vital amenities and cultivating a feeling of unity among drivers and passengers.

## Conclusion

The current research highlights the effect of transportation restrictions on health and resilience in Palestine, highlighting both direct and indirect impacts. Palestinians confront a myriad of obstacles while commuting for medical care, including checkpoints, road closures, and military interventions, resulting in delayed or deferred medical treatment and exacerbating preexisting health conditions [27, 28]. Conversely, transportation constraints also shape resilience, with Palestinians exhibiting remarkable fortitude in navigating transportation-related health challenges.

Our findings underscore the pivotal role of residence location in shaping health outcomes, with residents of camps and the “green line” area demonstrating higher levels of resilience [26]. Disparities in health outcomes between Palestinian towns and cities are exacerbated by obstacles and checkpoints, exacerbating socioeconomic, humanitarian, and health-related difficulties in areas such as Nablus, Jenin, and Tulkarm, thereby exacerbating inequality and social disruption.

Furthermore, our research highlights the critical influence of transportation means on health outcomes, with public transit proving more beneficial than private vehicles [29]. Resilient public transportation networks ensure uninterrupted access to basic services and foster a sense of community among residents, contributing to improved health outcomes.

Drawing upon a wealth of literature, our study elucidates the complex interplay between conflict situations, transportation restrictions, and individual well-being. Environmental and psychological factors, including resource scarcity, political violence, and attachment security, significantly influence resilience and vulnerability in conflict environments [17] The detrimental effects of limited access to healthcare due to movement restrictions are evident, underscoring the urgent need for interventions to mitigate their impact on Palestinian well-being.

Political violence perpetuates a cycle of humiliation and disruption, exacerbating psychological distress and compromising long-term health, economic, and psychological functioning [31, 31]. Resilience emerges as a crucial factor in promoting mental health and quality of life, with Palestinian cultures’ steadfastness, or “sumud,” leading to improved psychological well-being compared to that of their counterparts in more tranquil situations [32].

Moreover, our study reveals the nuanced nuances of attachment security, with secure attachment promoting greater resilience and adaptive functioning in the face of disasters and hardships [29]. Securely attached individuals exhibit strong emotional regulation and adaptable coping mechanisms, contributing to their ability to seek support from others in difficult situations.

In conclusion, our study contributes valuable insights into the multifaceted impacts of transportation restrictions on health and resilience in Palestine, offering implications for policy and interventions aimed at promoting well-being amidst adversity.

### Practical implications

The current research has underscored the need for substantiated reforms within the Palestinian health and transportation systems to develop transportation infrastructure and improve convenient access to essential healthcare services in Palestine. The government should invest in public transportation systems as well as community resilience programs to improve Palestinians’ lives and help them handle transportation-related health problems and challenges. Efforts to address security concerns, particularly in villages facing frequent settler attacks, are crucial. The key Palestinian sectors, including but not limited to the ministries of Health, Transportation, and Interior Affairs, are more than necessary to develop comprehensive policies and strategies to improve the deteriorating health system due to conflict and increasing threats to Israeli settlers.

### Limitations

Despite the key findings obtained, this research could have ignored other health determinants, including but not limited to individual lifestyles and behaviors as well as environmental factors, and may not adequately understand the complexity of overall well-being and health conditions in the territories occupied by Palestinians. Due to unique sociopolitical and geographical constraints, there may be limits to the generalizability of the findings to other populations or settings. Furthermore, self-reported data and biases may affect the accuracy and reliability of the results due to limitations of the methodological approach. It may be difficult to assess long-term impacts and causal pathways without a longitudinal approach. The study’s reliance on self-reported data may introduce bias due to respondents’ personal opinions and feelings of persecution under occupation, potentially overestimating the impact of limited mobility on health status. To mitigate this limitation, the authors could consider supplementing self-reported data with objective measures of health outcomes. Additionally, the timing and geographic scope of the data collection may influence the study’s findings. Seasonal variations, changes in conflict intensity, and shifts in healthcare service availability could impact the results. The areas included may not fully represent diversity within the Palestinian territories, limiting generalizability.

### Implications for future research

Prospective researchers could investigate other health determinants in Palestinian society, encompassing the nexus between transportation restrictions and further environmental, social, and economic factors. To evaluate the long-term effect of transportation restrictions on health outcomes, future research should consider carrying out longitudinal investigations.

## Data Availability

Data is provided within the supplementary information files.
